# A Unique Junctional Interface at Contact Sites Between the Endoplasmic Reticulum and Lipid Droplets

**DOI:** 10.3389/fcell.2021.650186

**Published:** 2021-04-08

**Authors:** Vineet Choudhary, Roger Schneiter

**Affiliations:** ^1^Department of Biotechnology, All India Institute of Medical Sciences (AIIMS), New Delhi, India; ^2^Department of Biology, University of Fribourg, Fribourg, Switzerland

**Keywords:** ER subdomains, lipid droplet, organelle biogenesis, membrane contact site, membrane trafficking, seipin, lipodystrophy, lipid storage disorders

## Abstract

Lipid droplets (LDs) constitute compartments dedicated to the storage of metabolic energy in the form of neutral lipids. LDs originate from the endoplasmic reticulum (ER) with which they maintain close contact throughout their life cycle. These ER–LD junctions facilitate the exchange of both proteins and lipids between these two compartments. In recent years, proteins that are important for the proper formation of LDs and localize to ER–LD junctions have been identified. This junction is unique as it is generally believed to invoke a transition from the ER bilayer membrane to a lipid monolayer that delineates LDs. Proper formation of this junction requires the ordered assembly of proteins and lipids at specialized ER subdomains. Without such a well-ordered assembly of LD biogenesis factors, neutral lipids are synthesized throughout the ER membrane, resulting in the formation of aberrant LDs. Such ectopically formed LDs impact ER and lipid homeostasis, resulting in different types of lipid storage diseases. In response to starvation, the ER–LD junction recruits factors that tether the vacuole to these junctions to facilitate LD degradation. In addition, LDs maintain close contacts with peroxisomes and mitochondria for metabolic channeling of the released fatty acids toward beta-oxidation. In this review, we discuss the function of different components that ensure proper functioning of LD contact sites, their role in lipogenesis and lipolysis, and their relation to lipid storage diseases.

## Introduction

**FIGURE 1:**
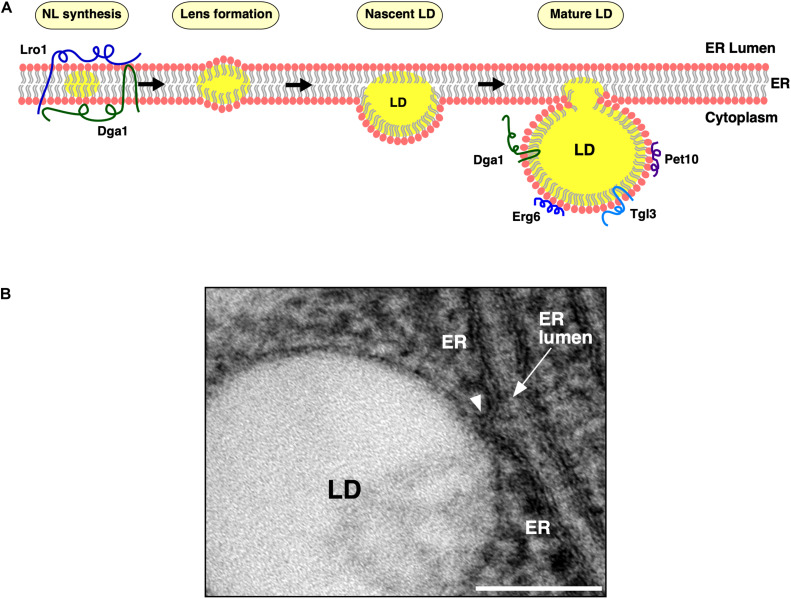
Lipid droplet (LD) biogenesis from the endoplasmic reticulum (ER) membrane. **(A)** Model of LD biogenesis. ER residential triacylglycerol-producing enzymes, Dga1 and Lro1, catalyze production of neutral lipids (NLs, shown in yellow) within the lipid bilayer of the ER membrane. Upon reaching a concentration threshold, NLs coalesce and form lens-like structures. NL lenses acquire more NLs, grow into nascent LDs, which then emerge toward the cytoplasm and further mature in their protein composition. Mature LDs remain connected to the cytoplasmic leaflet of the ER membrane at ER–LD contact sites. The TAG-synthase, Dga1, and the TAG-lipase, Tgl3, then translocate from the ER bilayer onto the LD monolayer of mature LDs. Finally, LD scaffolding proteins, such as the perilipin ortholog, Pet10, and the sterol biosynthetic enzymes, such as Erg6, localize to the periphery of mature LDs. **(B)** Morphology of the ER–LD junction. Ultrastructural visualization of the continuity between the ER bilayer membrane and the LD surface. ER, endoplasmic reticulum; LD, lipid droplet. Scale bar, 0.1 μm.

Lipid droplets (LDs) serve to store metabolic energy in the form of neutral lipids (NLs) such as triacylglycerols (TAG) and sterol esters (STE), generally known as fats. LDs are functionally and, to a large extent, also structurally conserved over a wide range of organisms including plants, fungi, and vertebrae. In all eukaryotes, NLs are synthesized by endoplasmic reticulum (ER)-residential acyltransferases. These enzymes, catalyze the conversion of either diacylglycerol (DAG) or free sterols (mostly ergosterol in yeast) to TAG and STE, respectively, by utilizing coenzyme A (CoA)-activated fatty acids as co-substrate (Walther et al., 2017). In yeast, Dga1 and Lro1 produce TAG, whereas Are1 and Are2 generate STE. Remarkably, NLs are dispensable for yeast since a quadruple mutant (*dga1*Δ *lro1*Δ *are1*Δ *are2*Δ) is viable but lacks the capacity to synthesize NLs and thus has no detectable LDs (Sandager et al., 2002). Mammals express two different diacylglycerol acyltransferase (DGAT) enzymes: a polytopic DGAT1 enzyme and an evolutionarily unrelated DGAT2 enzyme that is similar to yeast Dga1 and harbors a hairpin type of membrane topology (Stone et al., 2006; Walther et al., 2017). Upon reaching a threshold concentration of ∼3–5 mol% within the ER bilayer, newly synthesized NLs coalesce into ER-embedded NL lenses, grow in size by acquiring more NLs, and gradually transform into nascent LDs, which then emerge toward the cytoplasm, while remaining connected to the ER (Khandelia et al., 2010; Olzmann and Carvalho, 2019). Mature LDs thus have a unique architecture, a core of NLs enveloped by a phospholipid monolayer, which is continuous with the cytoplasmic leaflet of the ER membrane (Figures 1A,B). LDs maintain a close relationship with the ER, possibly throughout their life cycle (Figure 1B). A continuous exchange of proteins and lipids between these two compartments facilitates the dynamic remodeling of LDs in response to metabolic demand (Jacquier et al., 2011; Salo et al., 2016). In this review, we concentrate on the discussion of early steps of LD formation that are important for the proper formation of ER-LD junctions, focusing mostly on proteins that have initially been identified and characterized in *Saccharomyces cerevisiae*, but we will also provide an insight into possible differences in the process in mammalian cells. In addition, we will briefly cover contact sites between LDs and other organelles, particularly peroxisomes and mitochondria.

## The Endoplasmic Reticulum–Lipid Droplet Junction Forms a Unique Interface Between the Endoplasmic Reticulum Lipid Bilayer and Lipid Droplet Monolayer

Contact sites between organelles enable inter-organellar communication, including the transport of ions, lipids, and other small molecules. These membrane contacts are frequently established by molecular tethers, which bring two organelle membranes into nanometer distance of each other, as exemplified by the tricalbins, orthologs of the extended-synaptotagmins (E-Syts), which tether the ER to the plasma membrane (Manford et al., 2012). Mature LDs form extensive contacts with many organelles, including the ER, mitochondria, the Golgi, peroxisomes, and lysosomes/vacuoles (Barbosa and Siniossoglou, 2017; Schuldiner and Bohnert, 2017). ER–LD junctions are unusual as they are established during the biogenesis of LDs from the ER membrane, and they connect the ER bilayer to the LD monolayer via a continuous lipidic bridge between the two compartments (Figure 1B). The ER–LD junctions therefore are strikingly different from other contact sites between organelles delineated by bilayer membranes. This architecture at the ER–LD junction constitutes a barrier for ER residential integral membrane proteins and excludes them from the LD monolayer (Khaddaj et al., 2020). Early cryo electron microscopy (EM) studies have revealed that the cytoplasmic leaflet of the ER membrane is continuous with the LD surface, and freeze fracture EM suggested that the ER membrane forms a cup-like structure that holds the LD in place (Blanchette-Mackie et al., 1995; Robenek et al., 2006). Quantitative 3D-EM in mammalian cells indicates that LDs remain connected to the ER and that this connection is mediated by seipin, a protein that specifically localizes to ER–LD junctions (Salo et al., 2016). Proper formation of these ER–LD junctions requires a delicate interplay between locally enriched LD biogenesis factors and lipids to drive efficient LD formation.

## Lipid Droplets Are Formed From Endoplasmic Reticulum Subdomains That Contain Specific Proteins and Lipids

Previous work has identified several proteins that are important for early steps of LD formation. These include seipin (Fld1/Sei1 in yeast) (Szymanski et al., 2007; Fei et al., 2008); the lipin complex composed of Nem1/Spo7/Pah1 in yeast (Adeyo et al., 2011; Kwiatek et al., 2020); fat-storage-inducing transmembrane protein (FITM; Yft2 and Scs3 in yeast) (Kadereit et al., 2008); ACSL3, a key acyltransferase that activates fatty acids (Kassan et al., 2013); Pex30, an ER membrane-shaping protein (Joshi et al., 2018; Wang et al., 2018); and members of the perilipin (PLIN) family of LD scaffolding proteins (Pet10 in yeast) (Jacquier et al., 2013; Gao et al., 2017). Many of these proteins are ER-localized integral membrane proteins, including seipin, Nem1, Spo7, and FITM. Recently, using a conditional yeast mutant for the induction of *de novo* LD biogenesis, these factors have been shown to colocalize with each other to define discrete sites of LD biogenesis in the ER membrane from where they coordinate the ordered nucleation and growth of a droplet (Choudhary et al., 2020). ER membrane proteins that are not involved in LD biogenesis such as Sec63, a component of the protein translocon complex, are excluded from ER subdomains engaged in LD biogenesis (Choudhary et al., 2020). In the absence of LDs, some LD-localized proteins are unstable and become degraded through the ERAD (ER-associated protein degradation) quality control system (Ruggiano et al., 2016).

Spatial definition of LD biogenesis, however, does not only depend on specific proteins but also requires local enrichment of particular lipids, whose biochemical and biophysical properties promote the assembly of LD biogenesis factors (Ben M’barek et al., 2017; Choudhary et al., 2018, 2020; Choudhary and Schneiter, 2020; Santinho et al., 2020). Lipin (Pah1 in yeast) is a key enzyme that controls the bifurcation between membrane expansion and the production of storage lipids. Pah1 catalyzes the conversion of phosphatidic acid (PA) to diacylglycerol (DAG), which is then used by Dga1 and Lro1 to produce TAG and hence promote LD formation and growth (Kwiatek et al., 2020). Pah1 activity and its turnover are controlled by Nem1, the catalytic subunit of the Nem1-Spo7 phosphatase complex (Kwiatek et al., 2020). In mammalian cells, lipins are bifunctional proteins and also translocate to the nucleus where they act as a transcriptional coactivator to stimulate expression of genes involved in fatty acid oxidation (Reue and Wang, 2019).

Based on theoretical and experimental evidence, the formation of ER–LD junctions is affected by the intrinsic molecular curvature of ER phospholipids (Choudhary et al., 2018; Gao et al., 2019). While lipids that promote negative curvature such as DAG stabilize an ER embedded state of LDs, lipids that promote positive membrane curvature, such as lysophospholipids, will favor emergence of LDs toward the cytoplasm (Choudhary et al., 2018; Gao et al., 2019). In addition, the directionality of LD emergence from the ER, i.e., whether LDs bud toward the cytoplasm or the ER lumen, is affected by an asymmetric distribution and packaging of lipids at LD biogenesis sites, surface tension, membrane curvature properties, and lateral pressure (Chorlay et al., 2019; Gao et al., 2019). Even the acyl composition on ER phospholipids affects LD formation as lipids containing short chain and saturated fatty acids antagonize LD formation and instead promote NL accumulation in the ER (Hashidate-Yoshida et al., 2015; Cao et al., 2021; Zoni et al., 2021). *In vitro* studies with model membranes indicate that a reduction in surface tension through the accumulation of lysophospholipids promotes budding of small LDs (Ben M’barek et al., 2017). Artificial LDs embedded into giant unilamellar vesicles, for example, bud toward the side having higher coverage with phospholipids and proteins, resulting in LDs with a reduced surface tension (Chorlay et al., 2019). A continuous supply of phospholipids within the ER cytoplasmic leaflet is thus important to ensure emergence of LDs into the cytoplasm. When cells are grown in media containing oleic acid, a common naturally occurring fatty acid, which promotes NL formation in the ER, the capacity to supply sufficient phospholipids can become overwhelming, resulting in aberrantly enlarged ER–LD junctions and the emergence of LDs toward the ER lumen (Mishra et al., 2016; Chorlay et al., 2019). Failure of LDs to emerge from the ER toward the cytoplasm will impair the interaction of LDs with other organelles and abrogate the recruitment of cytoplasmic regulatory proteins and lipolytic enzymes to the LD surface, thereby impeding NL metabolism (Olzmann and Carvalho, 2019).

## Proteins That Define Endoplasmic Reticulum–Lipid Droplet Junctions

### Seipin

Seipin is the best-studied ER–LD junction protein. It is encoded by *BSCL2* in humans and *FLD1/SEI1* in yeast (Magre et al., 2001; Szymanski et al., 2007; Fei et al., 2008). Although the exact mode of action of seipin is not yet clear, recent studies have provided some mechanistic insights into the function of seipin. Seipin is an integral membrane protein of the ER that plays an essential role in LD formation and normal adipogenesis. Seipin contains short N- and C-terminal domains oriented toward the cytoplasm, two transmembrane domains, and a highly conserved large ER luminal domain (Szymanski et al., 2007; Cartwright and Goodman, 2012). The N-terminal domain of seipin is crucial for the initiation of LD formation in yeast, since deletion of this domain results in a seipin null phenotype (Cartwright et al., 2015). In humans, mutations in seipin result in lipodystrophy syndromes, a group of disorders that are characterized by a selective loss of adipose tissue (Magre et al., 2001), and seipinopathy, neurological disorders affecting motor neurons (Windpassinger et al., 2004; Cartwright and Goodman, 2012). Seipin localizes to ER–LD junctions in both yeast and mammalian cells (Szymanski et al., 2007; Fei et al., 2008; Salo et al., 2016; Wang et al., 2016). Absence of seipin from these sites results in growth-arrested LDs, manifesting in numerous tiny LDs, and a few supersized LDs, many of which are not functionally connected with the ER membrane, resulting in an altered LD surface proteome (Grippa et al., 2015; Salo et al., 2016; Wang et al., 2016).

In yeast, seipin forms a complex with Ldb16, a seipin partner protein with no identified mammalian homolog (Wang et al., 2014; Grippa et al., 2015). Ldb16 is an ER-localized integral membrane protein that interacts with seipin through its transmembrane helices (Wang et al., 2014). Deletion of either partner protein results in size heterogeneity of LDs. Expression of human seipin rescues this LD phenotype, suggesting that human seipin functionally complements the yeast seipin–Ldb16 complex (Wang et al., 2014).

**FIGURE 2:**
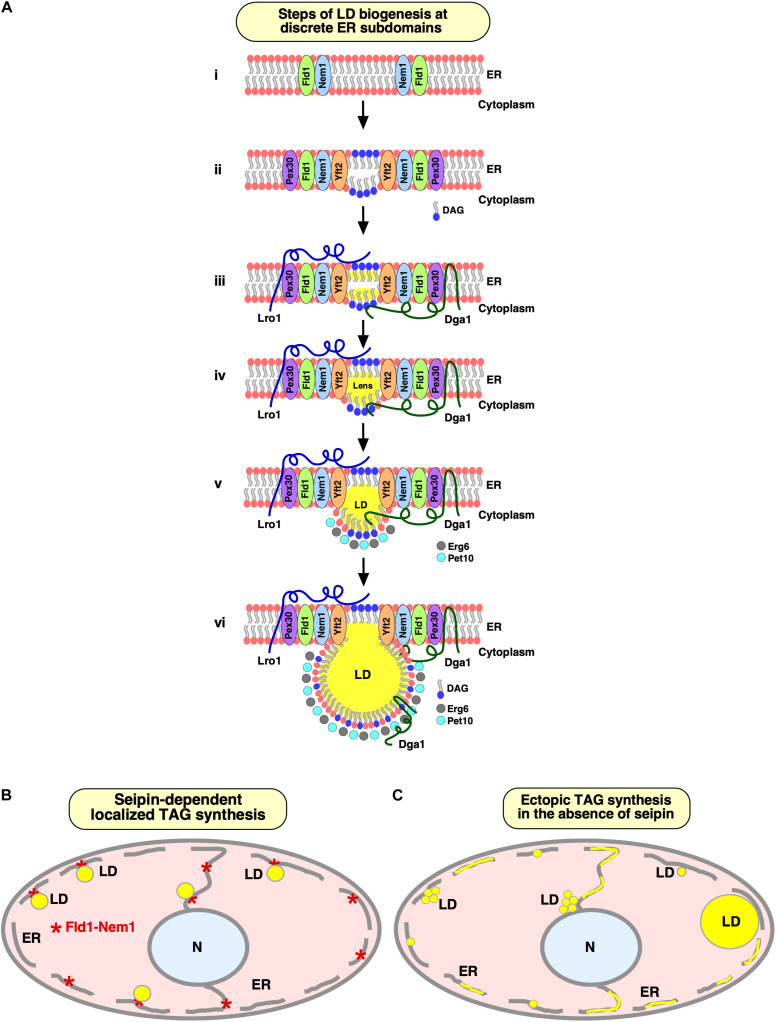
Specialized ER subdomains engaged in LD biogenesis. **(A)** Steps of LD biogenesis from discrete ER subdomains. (i) Seipin/Fld1 and Nem1 localize to discrete domains in the ER in LD-deficient yeast cells. Colocalization of Fld1 and Nem1 (Fld1/Nem1 sites) results in activation of Nem1 at these sites. (ii) The phosphatidate phosphatase, Pah1, gets activated and catalyzes production of DAG at Fld1/Nem1 sites. Locally enriched DAG then drives the recruitment of Yft2 and Pex30 to these sites. Deformation of the ER bilayer is facilitated by Pex30 to accommodate DAG and/or TAG. (iii, iv) Fld1/Nem1/Yft2/Pex30 containing ER subdomains become functional in recruiting the TAG-synthesizing enzymes: Dga1 and Lro1 to these sites, resulting in the localized production of neutral lipids (iii), and formation of neutral lipid lenses (iv). (v, vi) Neutral lipid lenses grow into nascent LDs (v). LD scaffolding proteins, such as Pet10, and sterol biosynthetic enzymes, such as Erg6, recognize nascent LDs and facilitate their emergence toward the cytoplasm (v), where they further mature (vi). Mature LDs remain connected to the ER via a hemi-membranous bridge at ER–LD contact sites to facilitate bidirectional transport of lipids and proteins between the two compartments in a dynamic fashion. This figure has been modified from 2020 Choudhary et al. Originally published in the *Journal of Cell Biology* (https://doi.org/10.1083/jcb.201910177). **(B,C)** Seipin facilitates localized droplet assembly. Cartoon depicting seipin-marked ER sites at which localized TAG synthesis drives formation of nascent LDs, which remain associated with the ER at their site of biogenesis **(B)**. Lack of seipin results in ectopic TAG synthesis throughout the ER and formation of a heterogenous population of tiny, large, and clustered LDs **(C)**.

Fld1 and Nem1 together define discrete ER subdomains from where LDs are being formed (Choudhary et al., 2020). These seipin/Nem1 subdomains become coenriched with DAG, as revealed by colocalization with a fluorescent ER-DAG sensor, and recruit additional LD biogenesis factors, such as FITM, Pex30, and the TAG-synthases, Dga1, and Lro1 to promote NL synthesis and formation of nascent LDs (Choudhary et al., 2020) (Figures 2A,B). Nascent LDs then undergo further growth and expansion via three distinct mechanisms: Synthesis of NLs by LD-localized acyltransferases (Sorger and Daum, 2002; Wilfling et al., 2013), flow of NLs from the ER through ER–LD junctions (Jacquier et al., 2011; Kassan et al., 2013; Wilfling et al., 2013), and transfer of NLs between adjacent LDs through a ripening process, i.e., transfer of TAG from smaller to larger LDs (Thiam et al., 2013; Salo et al., 2019). Mature LDs remain associated with seipin/Nem1 puncta in the ER (Figure 2B). Absence of any of these factors impedes droplet biogenesis and can result in ectopic TAG synthesis throughout the ER membrane and the formation of aberrant LDs (Choudhary et al., 2020) (Figure 2C).

Seipin preferentially enriches at ER tubules rather than ER sheets, owing to the higher curvature of the tubular ER domains, and thereby controls LD nucleation from these highly curved domains (Santinho et al., 2020). Such an enrichment of LD biogenesis factors at ER subdomains might modulate the local lipid composition, particularly that of DAG/TAG to promote TAG synthesis and/or control its nucleation by preventing the diffusion of NLs into the bulk of the ER membrane. Consistent with such a lipid gatekeeper function at ER–LD junctions, loss of seipin results in flow of NLs from tiny LDs via the ER membrane into large LDs causing the pronounced heterogeneity in LD size (Salo et al., 2019). In addition, seipin has been shown to regulate PA levels at ER–LD junctions (Han et al., 2015; Wolinski et al., 2015; Pagac et al., 2016) and to act as a scaffold for lipid-synthesizing enzymes (Talukder et al., 2015). ER–LD junctions have been analyzed by EM in various cell types, and they appear to be conserved (Jacquier et al., 2011; Wilfling et al., 2013; Ohsaki et al., 2016; Salo et al., 2016; Wang et al., 2016; Romanauska and Kohler, 2018). Interestingly, besides cytoplasmic LDs, cells can also generate LDs in the nucleoplasm. Formation of these nucleoplasmic LDs (nLDs) depends on TAG producing enzymes that are localized to the inner nuclear membrane (INM) (Romanauska and Kohler, 2018). More recently, seipin has been shown to restrain the formation of nLDs, and seipin-deficient yeast mutants show dramatic increase in nLD formation (Cartwright et al., 2015; Romanauska and Kohler, 2018). Although seipin appears to be absent from the INM, deficiency of seipin results in elevated levels of PA in the INM and an increase in lipin-1 expression, which is likely to promote the formation of nLDs (Soltysik et al., 2021).

On the other hand, hepatocytes generate abundant nLDs that are derived from apolipoprotein B (ApoB)-free luminal LDs, a process that is dependent on microsomal triglyceride transfer protein (MTP) (Soltysik et al., 2019). Under conditions of ER stress, ApoB levels are reduced due to co- and posttranslational degradation (Ginsberg and Fisher, 2009), whereas MTP levels are maintained (Qiu et al., 2005). This lack of ApoB-dependent NL secretion promotes the accumulation of ApoB-free luminal LDs, which grow inside the ER lumen and eventually disintegrate the INM leading to nLD formation (Soltysik et al., 2019). Thereby, nLD formation in hepatocytes may contribute to non-alcoholic fatty liver disease and its associated complications (Seebacher et al., 2020).

Seipin assembles into a ring-shaped oligomeric structure as revealed by recent cryo-EM studies of the luminal domain of human (undecamers) and fly (dodecamers) Seipin (Sui et al., 2018; Yan et al., 2018). Each monomer contains a hydrophobic helix that is apposed toward the ER bilayer, and a ß-sandwich domain with a structural similarity to lipid-binding proteins, such as Niemann-Pick type C2 (NPC2), a cholesterol-binding protein (Sui et al., 2018). This ER luminal ß-sandwich domain has affinity toward anionic phospholipids such as PA (Yan et al., 2018). Based on these findings, seipin was proposed to scan the ER membrane for small TAG lenses, where it could get anchored and facilitate further growth of LDs by transferring newly synthesized TAG from the ER membrane into the growing LDs (Sui et al., 2018). Furthermore, the seipin complex at the base of ER–LD junctions might create a selectivity barrier and thereby uncouples the lipid composition of these ER subdomains from the bulk of the ER. Based on molecular dynamic simulations, seipin has recently been proposed to directly interact with, and thereby enrich, TAG and DAG within its ring-shaped structure (Prasanna et al., 2020; Zoni et al., 2020). Hence, seipin might function as a lipid transporter and/or gatekeeper to locally regulate lipid levels at ER–LD junctions.

### Lipid Droplet Organization and Promethin/Lipid Droplet Assembly Factor 1

**FIGURE 3:**
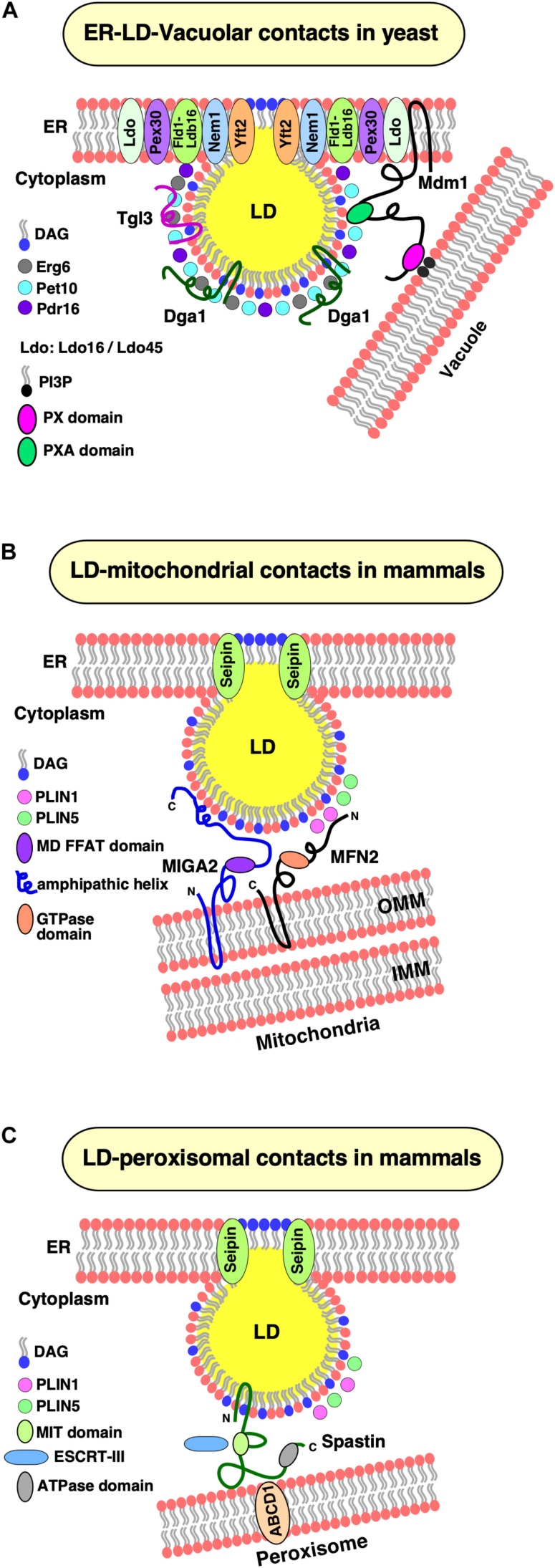
LD contacts with cellular organelles. **(A)** Establishment of ER–LD–vacuolar contacts in yeast. Yeast cells accumulate LDs at nuclear–vacuolar junctions (NVJ) during starvation. Under these conditions, components of the lipid droplet organization (Ldo) machinery (Ldo16 and Ldo45) coenrich with the Fld1–Ldb16 complex at ER–LD contact sites. Yeast Mdm1 also localizes at these sites and bridges three organelles together, the ER, LDs, and the vacuole. The PX domain of Mdm1 interacts with PI3P in the vacuolar membrane, whereas its PXA domain is sufficient for association with the LD surface. The PXA domain binds free fatty acids (FFA) and recruits the fatty acid synthase, Faa1, to generate a local pool of activated fatty acids thereby spatially regulating neutral lipid synthesis and growth of LDs. **(B)** LD–mitochondrial contacts. Under lipolytic stimulation of mammalian cells, LDs and mitochondria display extensive contact formation to facilitate transfer of metabolites, particularly fatty acids. In brown adipocytes, Mitofusin 2 (MFN2), an outer mitochondrial membrane (OMM) protein interacts with Perilipin 1 (PLIN1) on LDs and tethers the two organelles together. Another perilipin family member, PLIN5 is enriched at LD–mitochondrial contacts and possibly interacts with another partner protein at the OMM. In white adipocytes, Mitoguardin 2 (MIGA2), an OMM protein interacts with LDs with its C-terminal amphipathic domain. The middle domain of MIGA2 contains a FFAT motif that mediates tri-organellar contact formation (not shown) between the ER, mitochondria, and LDs. **(C)** LD–peroxisomal contacts. Spastin, mediates contacts between LDs and peroxisomes. The N-terminal hairpin domain of Spastin anchors it to the LD monolayer and an ATPase domain at its C-terminus that interacts with the peroxisomal ABCD1 transporter. The middle region of spastin contains a microtubule interacting and trafficking (MIT) domain that binds to two ESCRT-III proteins, which are required for transfer of fatty acids between LDs and peroxisomes.

The lipid droplet organization (Ldo) proteins, Ldo16, and its splice variant Ldo45 were recently identified as genetic interactors of seipin (Eisenberg-Bord et al., 2018; Teixeira et al., 2018). Ldo16 and Ldo45 play important roles in regulating LD biogenesis at a spatially confined ER domain adjacent to the nuclear–vacuolar junction (NVJ), a large contact site between the nuclear ER and the vacuole, during nutritional stress (Eisenberg-Bord et al., 2018). Ldo45 promotes TAG accumulation and hence proliferation of LDs, whereas Ldo16 is necessary for efficient lipophagy, LD consumption by autophagy (Teixeira et al., 2018). Surprisingly, overexpression of Ldo45 leads to clustering of LDs, mimicking the seipin deletion phenotype, suggesting an antagonistic role of Seipin and Ldo45 in regulating LD formation and maturation (Eisenberg-Bord et al., 2018). Ldo45 facilitates the targeting of Pdr16, a Sec14-like phosphatidylinositol transfer protein, to LDs at NVJ (Eisenberg-Bord et al., 2018) (Figure 3A). This family of proteins has previously been shown to localize at organelle contact sites (Selitrennik and Lev, 2016). Surprisingly, Pdr16 is mislocalized in cells lacking Ldo45; however, double deletion of Ldo45 together with Seipin, rescues the targeting of Pdr16 to LDs (Eisenberg-Bord et al., 2018). However, the precise mechanism of Pdr16 targeting to a subpopulation of LDs, and its role in LD metabolism, are not yet completely understood.

Promethin/lipid droplet assembly factor 1 (LDAF1) is a remote homolog of Ldo45 and a seipin partner protein identified in humans and widely conserved across species (Eisenberg-Bord et al., 2018; Chung et al., 2019). In mammalian cells, seipin collaborates with promethin/LDAF1 to define discrete ER sites of LD biogenesis (Chung et al., 2019). Promethin/LDAF1 localizes to LDs upon fatty acid supplementation, and copurifies with seipin, suggesting that these two proteins form a complex (Castro et al., 2019). In an independent study, promethin/LDAF1 was identified as an interactor with the conserved hydrophobic helix of seipin (Chung et al., 2019). During oleate-induced LD biogenesis, seipin remains localized at ER–LD junctions, whereas LDAF1 loses its association with seipin foci in the ER, and moves onto the periphery of expanding LDs. These findings suggest that TAG accumulation results in disruption of the interaction between promethin/LDAF1 and seipin and that the protein can move from a bilayer onto a monolayer membrane (Chung et al., 2019). In cells devoid of LDs, most of the LDAF1 puncta colocalize with seipin foci, while free LDAF1 and seipin foci also coexist, but *de novo* LDs begin to form at sites that contain both LDAF1 and seipin, suggesting that both proteins play a key role in defining ER sites of LD biogenesis (Chung et al., 2019). In agreement with this, relocalization of LDAF1 to ER–plasma membrane contacts results in localization of seipin at these ectopic sites from where LDs can then form *de novo* (Chung et al., 2019). Protease protection assays indicate that both N- and C-termini of LDAF1 are oriented toward the cytoplasm, suggesting a hairpin type of topology with a central hydrophobic domain that is sufficient for both colocalization with seipin and association with the LD monolayer (Chung et al., 2019). Analyses of copurifying lipids indicate that the promethin/LDAF1–seipin complex, but not seipin alone, binds TAG. Interestingly, absence of seipin also results in loss of promethin/LDAF1, suggesting that previous studies analyzing seipin deficiency may, in fact, represent conditions where both partner proteins, seipin and LDAF1, were missing (Chung et al., 2019).

### Fat-Storage-Inducing Transmembrane Proteins

FITM proteins constitute an evolutionarily conserved family of polytopic ER-localized transmembrane proteins (Kadereit et al., 2008). Mammals express two FITM isoforms: FITM1 is mainly expressed in skeletal muscles, while FITM2 is expressed in most tissues. FITM2 plays a major role in the differentiation of preadipocytes into mature adipocytes. It is functionally regulated by peroxisome proliferator-activated receptor gamma (PPARγ), and controls the accumulation of TAG within LDs (Kadereit et al., 2008). While overexpression of FITM2 in 3T3-L1 adipocytes and in mouse liver *in vivo* results in accumulation of TAG-rich LDs, its deficiency leads to a decrease in both size and number of LDs (Kadereit et al., 2008). Purified FITM2 binds both DAG and TAG *in vitro*, but the protein itself has no TAG biosynthetic activity (Gross et al., 2011). Based on the observation that FITM proteins become enriched at ER–LD junctions upon LD formation, it has been suggested that they promote the condensation/sequestration of TAG in the ER bilayer and/or packaging of TAG into LDs (Kadereit et al., 2008; Gross et al., 2011).

The knockout of FITM2 in adipose tissue in mice results in lipodystrophy and insulin resistance (Miranda et al., 2014). In humans, a homozygous nonsense mutation in FITM2 was identified in a family with Siddiqi syndrome, characterized by deafness–dystonia syndrome, delayed development, and regression of motor skills, dystonia, ichthyosis-like features, signs of sensory neuropathy, and low body mass index (Zazo Seco et al., 2017). Interestingly, RNAi-mediated knockdown of the sole FITM2 in *Drosophila melanogaster* recapitulated many of the characteristic features of the human phenotype, suggesting a link between the mutation in FITM2 and the phenotypic syndrome (Zazo Seco et al., 2017). The causal relation between the defect in TAG formation or storage and these neurological syndromes in organisms lacking FITM function, however, remains to be characterized.

FITM2 is more widely conserved than FITM1, since *Drosophila*, *Caenorhabditis elegans*, and *Saccharomyces cerevisiae* have only orthologs of FITM2. Budding yeast even has two FITM2 homologs, Yft2 and Scs3. Yeast FITM2 mutants have defects in ER membrane homeostasis, and *Scs3* mutant cells are auxotrophic for inositol (Hosaka et al., 1994; Moir et al., 2012). Lack of FITM2 proteins in yeast results in the formation of LDs that emerge toward the ER lumen instead of the cytoplasm, suggesting that these proteins are required to promote proper budding of nascent LDs from the ER (Choudhary et al., 2015). This function of FITM2 is conserved as depletion of FITM2 in human 3T3-L1 fibroblasts, and lack of the sole FITM2 in *C. elegans* results is a similar phenotype (Choudhary et al., 2015, 2016). Remarkably, deletion of FITM2 in *C. elegans* and mice is lethal, indicating that FITM2 fulfills a *hitherto* uncharacterized but essential function in lipid metabolism (Choudhary et al., 2015, 2016; Goh et al., 2015).

Sequence alignment suggests that FITM2 proteins could harbor a lipid phosphatase/phosphotransferase activity on the ER luminal side, and mutations of residues that are conserved among lipid phosphatases affect FITM2 function *in vivo* (Hayes et al., 2017). Consistent with this role, FITM2 becomes transiently enriched at LD biogenesis sites, where it may play an important role in regulating DAG levels, a critical prerequisite for proper LD formation (Adeyo et al., 2011; Choudhary et al., 2018). More recently, FITM2 was shown to harbor acyl-CoA diphosphatase activity and to hydrolyze fatty acyl-CoA to yield acyl 4′-phosphopantetheine (Becuwe et al., 2020). This enzymatic activity of FITM2 is important in maintaining proper structure of the ER membrane as lack of FITM2 results in the formation of ER whorls, and impacts both ER and lipid homeostasis, but it is presently not clear how exactly the lack of this enzymatic activity manifests in the many different phenotypes associated with mutations in FITM (Becuwe et al., 2020).

### Pex30/MCTP

Pex30, a membrane-shaping protein has recently been shown to be important for proper LD formation. Pex30 contains an N-terminal reticulon homology domain (RHD), a hairpin-type membrane domain similar to that present in the reticulon family of ER-shaping proteins, and a C-terminal Dysferlin (DysF) domain (Joshi et al., 2016). Similar to reticulons, Pex30 tubulates the ER membrane. However, unlike the abundantly expressed reticulons, which show uniform ER distribution, Pex30 is a low abundant protein that localizes to discrete ER subdomains from which nascent LDs and pre-peroxisomal vesicles originate (Joshi et al., 2016, 2018). Pex30 remains associated with mature LDs at ER–LD junctions and does not localize to ER exit sites at which COPII-coated vesicles form (Joshi et al., 2016). Deletion of Pex30, on the other hand, results in a significant delay in the rate of LD biogenesis, proliferation of ER membranes, and defects in peroxisome biogenesis, suggesting a shared common mechanism in the biogenesis of these two organelles (Joshi et al., 2018; Wang et al., 2018; Santinho et al., 2020). Dysferlin containing proteins in higher eukaryotes play an important role in the repair of damaged membranes due to mechanical stress as occurs in skeletal muscle cells, giving rise to muscular dystrophies, suggesting that Pex30 may harbor similar membrane repair properties (Glover and Brown, 2007). These properties are possibly important for seipin function and probably in the repair of ER membrane at sites where LDs and peroxisomes form.

The human homolog of Pex30, multiple C2 domain containing transmembrane protein (MCTP), also contains an RHD (Joshi et al., 2018). While mammals express two MCTP isoforms, worms and flies have only one MCTP (Shin et al., 2005). Similar to Pex30, MCTP2 is a low abundant protein that localizes to ER subdomains at which nascent LDs are born and associates with peroxisomes in a dynamic fashion (Joshi et al., 2018). The curvature-inducing property of Pex30/MCTP2 might be important to deform the ER bilayer at LD biogenesis sites to accommodate DAG and/or TAG. Moreover, Pex30 colocalizes with other LD biogenesis factors, such as seipin and Nem1, at DAG-enriched ER subdomains (Joshi et al., 2018). Interestingly, in cells lacking seipin, Pex30 is mislocalized to a single punctum (Joshi et al., 2018; Wang et al., 2018). It is possible that Pex30 directly interacts with seipin, and absence of seipin from ER subdomains is likely to destabilize Pex30 localization. Remarkably, simultaneous deletion of both Seipin and Pex30 results in strong synergistic growth defect, impaired LD formation, and elevated DAG levels, which antagonizes LD emergence, resulting in TAG accumulation throughout the ER membrane (Joshi et al., 2018; Wang et al., 2018). These growth and LD emergence defects in double mutant cells lacking both *Pex30* and seipin can be rescued by manipulating the lipid composition of the ER by deletion of the cholinephosphate cytidylyltransferase, Pct1, the rate-limiting enzyme of phosphatidylcholine biosynthesis through the Kennedy pathway, thereby reducing phosphatidylcholine, phosphatidylinositol, and DAG levels but increasing TAG levels (Wang et al., 2018).

Many proteins that dually target both the ER and LDs contain hydrophobic domains that adopt a V-shaped helical hairpin topology. How these hairpin-containing proteins are targeted to the LD surface, however, is not well understood. However, one such protein, UBX8, is localized to the ER with the help of two peroxisomal proteins. UBX8 is first captured by the soluble cytoplasmic protein Pex19, and subsequently inserted into the ER membrane at subdomains containing Pex3, from where it then translocates to the LD monolayer (Schrul and Kopito, 2016). Thus, Pex30-marked ER subdomains could mediate inter-organelle trafficking of proteins/lipids both between ER–LD and ER–LD–peroxisome contacts.

### Mdm1/Sortin Nexin 14

Mdm1 in yeast and its human ortholog Snx14 have recently been uncovered as molecular tethers at ER–LD junctions, providing novel insights into how these unique junctions are established and function in regulating LD biogenesis in response to metabolic demands (Datta et al., 2019; Hariri et al., 2019). Under starvation, LDs accumulate at spatially confined ER domains adjacent to the NVJ (Hariri et al., 2018). Mdm1 is a key NVJ component that is required for accumulation of LDs at NVJ during starvation and functions as a triorganellar molecular tether that connects the ER with LDs and the vacuole, thereby facilitating LD degradation (Hariri et al., 2018). Mdm1 is anchored in the ER via its N-terminal integral membrane domain, which is necessary and sufficient for targeting Mdm1 to LD biogenesis sites, and it targets the vacuolar membrane via a phosphatidylinositol 3-phosphate-binding Phox (PX) domain (Hariri et al., 2019). A third domain within Mdm1, termed PX-associated (PXA) domain, is sufficient for LD targeting; hence, Mdm1 can physically link the three compartments, the ER, LDs, and the vacuolar membrane (Hariri et al., 2019) (Figure 3A).

Mutations in the human ortholog of Mdm1, Snx14, lead to spinocerebellar ataxia autosomal recessive 20 (SCAR20) disease, which is characterized by intellectual disability, cerebellar atrophy, ataxia, and speech disorders (Thomas et al., 2014). Like Mdm1, Snx14 is an ER protein that relocalizes to ER–LD junctions upon oleate addition (Datta et al., 2019). Snx14 is anchored in the ER via its amino-terminal integral membrane domain and contains an amphipathic helix in the C-terminus, termed the C-Nexin domain, which is necessary for binding LDs in trans (Datta et al., 2019). Consistent with an ER–LD tethering function, overexpression of Snx14 dramatically increases ER–LD junctions, whereas a lack of Snx14 reduces these junctions (Datta et al., 2019). Though Mdm1 and Snx14 have the same domain organization, the PX domain in Snx14 lacks the residues required for phosphoinositide binding (Mas et al., 2014), consistent with the fact that NVJ contacts are absent in mammalian cells. Remarkably, both Mdm1 and Snx14 were found to cooperate with fatty acyl-CoA synthetases to locally increase concentrations of activated fatty acids for the formation of TAG at ER–LD junctions. Thus, apart from acting as tethers between organelles, these proteins could also provide a scaffold for the assembly of enzymes to channel the flux of metabolites toward energy storage. Absence of Mdm1 sensitizes cells to fatty acid-induced lipotoxicity, suggesting a key role in lipid homeostasis, whereas Snx14 mutants display gross LD morphology defects, implying that Snx14 is required for the formation of functional LDs. It will be interesting to determine whether Mdm1 and Snx14 interact with other LD biogenesis factors at ER–LD junctions to coordinate lipogenesis with LD expansion.

### Ras-Related Protein Rab18

Rab18 belongs to the small GTPases family, which regulates membrane trafficking. Rab18 shows diverse intracellular localization and is found both at the ER and on LDs. Rab18 functions as a common mediator of lipogenesis and lipolysis in adipocytes (Pulido et al., 2011), and has been implicated in mediating the formation of ER–LD junctions (Ozeki et al., 2005). In agreement with a tethering function, overexpression of Rab18 greatly enhances ER–LD junctions possibly by decreasing Perilipin 2 levels on LDs (Ozeki et al., 2005). Rab18 interacts with the ER-associated NAG-RINT1-ZW10 (NRZ) tethering complex and their associated SNARES, resulting in the recruitment of ER to LDs and the formation of direct ER–LD junctions thereby promoting lipogenesis and LD growth (Xu et al., 2018). Even though the Rab18–NRZ complex functions to tether LDs to the ER, this function of Rab18 is not essential for maintenance of LD growth in all cell types (Jayson et al., 2018). Another partner protein of Rab18 is the double zinc finger FYVE domain-containing protein 1 (DFCP1), lack of which leads to decreased ER–LD junctions, whereas overexpression of DFCP1 results in increased junction formation (Li et al., 2019). Physiologically, mutations in Rab18 manifests in Warburg Micro syndrome, a collection of neurodevelopmental disorder characterized by optical atrophy, microcephaly, intellectual disability, and hypogenitalism (Bem et al., 2011; Dejgaard and Presley, 2019).

## Lipid Droplets Interact With Mitochondria and Peroxisomes

### Lipid Droplet–Mitochondrial Contacts

Upon activation of lipolysis, fatty acids that are released from LDs are oxidized by either mitochondria or peroxisomes to generate precursors for mitochondrial oxidative phosphorylation and energy production (Poirier et al., 2006). LDs thus form extensive contacts with these organelles to facilitate metabolite channeling of free fatty acids (Bohnert, 2020). In tissues that have a high-energy demand such as heart, skeletal muscle, brown adipocytes, and hepatocytes, pronounced contacts between LDs and mitochondria are observed (Wang et al., 2013; Boutant et al., 2017). These contact areas expand dramatically during starvation (Nguyen et al., 2017) or during endurance training of athletes (Shaw et al., 2008).

The factors that tether LDs and mitochondria are not yet fully characterized. Recently, tethering between the LD scaffolding protein Perilipin 1 (PLIN1) and Mitofusin 2 (MFN2), a mitochondrial outer membrane protein, has been observed in brown adipocytes (Boutant et al., 2017) (Figure 3B). Consistent with a tethering function during active lipolysis in brown adipocytes, PLIN1 co-precipitates with MFN2, and cells lacking MFN2 show reduced LD–mitochondrial contacts, impaired respiratory capacity, and reduced response to adrenergic stimulation (Boutant et al., 2017). Adipose-specific MFN2 knockout mice are protected from high-fat diet-induced insulin resistance and hepatic steatosis (Seebacher et al., 2020), suggesting that LD–mitochondrial interactions affect whole-body energy homeostasis (Boutant et al., 2017). Another perilipin family member, PLIN5, also localizes at LD–mitochondrial contacts (Gemmink et al., 2018) and is upregulated in oxidative tissues (Yamaguchi et al., 2006; Vigelso et al., 2016). However, its interacting mitochondrial partner protein remains yet to be identified.

Recently, a novel tether at LD–mitochondrial contacts containing Mitoguardin 2 (MIGA2) has been identified in white adipocytes (Freyre et al., 2019). Consistent with a tethering function of MIGA2, its overexpression in adipocytes greatly enhances LD–mitochondrial as well as ER–mitochondrial contact formation (Freyre et al., 2019). Overexpression of MIGA2 in cells that display very little LD–mitochondrial contacts, such as in COS7 cells, induces formation of extensive LD–mitochondrial contacts, suggesting that MIGA2 is sufficient to promote tethering of LD and mitochondria (Freyre et al., 2019). MIGA2 contains two N-terminal transmembrane domains that anchor the protein to the outer mitochondrial membrane, a middle domain, and a C-terminal amphipathic domain, which interacts with the LD surface (Freyre et al., 2019) (Figure 3C). Surprisingly, a truncated version of MIGA2 lacking both transmembrane domains, is unable to localize to LDs, despite the presence of the amphipathic domain (Freyre et al., 2019). Remarkably, the middle domain of MIGA2 contains a conserved peptide sequence called “two phenylalanines in an acidic tract” (FFAT) (Murphy and Levine, 2016), which binds VAMP-associated proteins (VAPs), a conserved family of integral ER membrane proteins, which mediate contact site formation between the ER and many different organelles, including the plasma membrane, peroxisomes, and the Golgi apparatus (Lev et al., 2008). MIGA2 thus facilitates tethering between three organelles, LDs, mitochondria, and the ER (Freyre et al., 2019). Importantly, lack of MIGA2 in preadipocytes results in impaired TAG synthesis, defects in LD growth, and blocks their differentiation into mature adipocytes, suggesting a crucial role of MIGA2 in coordinating and *de novo* lipogenesis in mitochondria to TAG production in the ER (Freyre et al., 2019). How exactly this coordination is achieved is presently not well understood, but mitochondria that are bound to LDs have unique bioenergetics, composition, and dynamics to support LD expansion (Benador et al., 2018).

### LD–Peroxisomal Contacts

As with the mitochondria, extensive contacts between LDs and peroxisomes have frequently been observed; however, little is known about the tethering components that establish these contacts (Novikoff et al., 1980; Binns et al., 2006; Thazar-Poulot et al., 2015). In yeast cells cultivated in the presence of oleic acid, which not only promotes rapid expansion of LDs but also induction of peroxisomes, extensive contacts between peroxisomes and LDs were observed (Binns et al., 2006). LDs isolated from such cells contained enzymes required for beta-oxidation of fatty acids, suggesting metabolic coupling between NL lipolysis in LDs and peroxisomal degradation of fatty acids. Consistent with such a metabolic coupling, free fatty acids accumulate on LDs in mutants defective in fatty acid beta-oxidation (Binns et al., 2006).

In mammalian cells, a tether complex comprised of the M1 spastin protein, which localizes to the LD surface, and the peroxisomal fatty acid transporter ATP-binding cassette subfamily D member 1 (ABCD1) (Figure 3C) has recently been uncovered (Chang et al., 2019). M1 spastin contains an N-terminal hydrophobic hairpin domain, which anchors it to the LD monolayer, a microtubule interacting and trafficking (MIT) domain, that selectively binds two ESCRT-III (endosomal sorting complex required for transport III) proteins, and a C-terminal AAA-ATPase domain (Chang et al., 2019). Localization of ESCRT-III at LD–peroxisomal contacts is crucial for fatty acid transfer, suggesting that ESCRT-III proteins promote fatty acid mobilization possibly by deformation of the LD membrane (Chang et al., 2019). Consistent with a role in tethering, overexpression of M1 spastin results in extensive LD–peroxisomal contacts in a microtubule-independent manner (Chang et al., 2019). Remarkably, M1 Spastin lacking the ATPase domain is still able to localize to LDs; however, it is unable to induce LD–peroxisomal contact formation (Chang et al., 2019). Spastin has been implicated in membrane remodeling and microtubule dynamics (Lumb et al., 2012). Mutations in spastin result in autosomal-dominant hereditary spastic paraplegia disease. Similarly, mutations in the peroxisomal fatty acid transporter protein, ABCD1, are associated with X-linked adrenoleukodystrophy and also manifest neurological symptoms such as spasticity (Morita and Imanaka, 2012). ABCD1 co-precipitates with Spastin or with its PXI domain alone, suggesting that these two proteins form a complex (Chang et al., 2019). Consistent with this, lack of ABCD1 abolishes LD-peroxisomal contacts, which cannot be rescued by overexpression of M1 Spastin, implying that the two partner proteins must be present for functional LD–peroxisomal contact formation (Chang et al., 2019). In agreement with this, overexpression of Spastin, enhances transfer of fatty acids via LD-peroxisomal contacts as revealed by pulse-chase experiments with a fluorescent fatty acid analog (Chang et al., 2019).

Taken together, contacts between LDs, on the one hand, and either mitochondria or peroxisomes, on the other hand, provide a novel means to channel the flux of metabolic intermediates and thereby integrate spatially segregated biochemical pathways. At the same time, these organellar contacts may play an essential role in controlling the progression of differentiation (Freyre et al., 2019). Alteration in LD contact formation due to mutations in molecular tethers are associated with different hereditary neurodevelopmental, neurodegenerative, and metabolic diseases (Herker et al., 2021). However, the exact molecular mechanism of the role of these LD contact site proteins in the etiology of these diseases is not well understood. Moreover, various intracellular pathogens, including bacteria, viruses, and parasites, are known to hijack the host LD machinery and induce dramatic rearrangement of LD contacts with cellular organelles for their own benefit and promote novel contact formation between LDs and the pathogen’s own replication organelle (Herker et al., 2021).

## Conclusion and Future Perspectives

Major advancements have been made over the past decade in unraveling components that are important for proper formation of LDs, thereby identifying proteins that localize to ER–LD contact sites. Formation of LDs from highly specialized ER subdomains is critical for both the biogenesis of LDs and their functional association with the ER membrane. In addition, contacts between LDs and other fatty acid-metabolizing organelles, particularly peroxisomes and mitochondria, are crucial to coordinate lipogenesis and lipolysis with the overall energy demand, both at cellular and organismal levels. This integration is likely complex and at the route of many phenotypes associated with the metabolic syndrome or mutations in individual components. Accordingly, many important questions still remain open: How is the formation of these contact sites regulated? How exactly do dysfunctional contact sites manifest in human pathologies such as lipodystrophy and/or neurological diseases? A better understanding of the nature of these contact sites and the characterization of the role of the individual components within these junctions are likely to bring novel insights into the etiology of lipid storage diseases, lipid homeostasis, and its integration with energy metabolism and body weight regulation.

## Author Contributions

VC contributed to the manuscript writing (initial draft) and figure preparation. VC and RS contributed to the manuscript editing and finalizing. Both authors contributed to the article and approved the submitted version.

## Conflict of Interest

The authors declare that the research was conducted in the absence of any commercial or financial relationships that could be construed as a potential conflict of interest.

## Abbreviations

ABCD 1: ATP-binding cassette subfamily D member 1
ATP: adenosine triphosphate
ApoB: apolipoprotein B
DAG: diacylglycerol
DFCP1: double zinc finger FYVE domain-containing protein 1
DGAT: diacylglycerol acyltransferase
EM: electron microscopy
ER: endoplasmic reticulum
ERAD: ER-associated protein degradation
ESCRT-III: endosomal sorting complex required for transport III
E-Syts: extended-synaptotagmins
FITM: fat-storage-inducing transmembrane proteins
FFAT: two phenylalanines in an acidic tract
INM: inner nuclear membrane
LDs: lipid droplets
LDAF1: lipid droplet assembly factor 1
Ldo: lipid droplet organization
MCTP: multiple C2 domain containing transmembrane protein
MFN 2: Mitofusin 2
MIGA 2: Mitoguardin 2
MIT: microtubule interacting and trafficking domain
MTP: microsomal triglyceride transfer protein
NL: neutral lipids
nLDs: nucleoplasmic LDs
NPC2: Niemann-Pick type C2
NVJ: nuclear-vacuolar junction
NRZ: NAG-RINT1-ZW10
PA: phosphatidic acid
PPAR: peroxisome proliferator-activated receptor
PLIN: perilipin
PX: phosphatidylinositol 3-phosphate-binding Phox
PXA: PX-associated
PXI: peroxisome-interacting motif
Rab18: Ras-related protein 18
RHD: reticulon homology domain
SCAR20: spinocerebellar ataxia autosomal recessive 20
STE: sterol esters
Snx: sortin nexin
TAG: triacylglycerol
VAPs: VAMP-associated proteins.
